# Different levels of glycosylated hemoglobin influence severity and long-term prognosis of coronary heart disease patients with stent implantation

**DOI:** 10.3892/etm.2014.2128

**Published:** 2014-12-11

**Authors:** JING WANG, GAOLIANG YAN, YONG QIAO, DONG WANG, GENSHAN MA, CHENGCHUN TANG

**Affiliations:** 1 Department of Cardiology, Zhongda Hospital of Southeast University Medical School, Nanjing, Jiangsu 210009, P.R. China; 2 Department of Cardiology, Huai’an First People’s Hospital, Nanjing Medical University, Huai’an, Jiangsu 223001, P.R. China

**Keywords:** glycosylated hemoglobin, coronary artery disease, Gensini score, major adverse cardiac events

## Abstract

The aim of this study was to investigate the correlation between glycosylated hemoglobin (HbA1c) levels and the severity and long-term prognosis of coronary heart disease (CHD) with stent implantation. A total of 2,825 consecutive patients with stent implantation were stratified into three groups based on the levels of HbA1c: Low HbA1c group (group A, HbA1c ≤5.9% or 41 mmol/mol; n=1,035), moderate HbA1c group (group B, 5.9%< HbA1c <6.8% or 41< HbAlc <51 mmol/mol; n=1,025) and high HbA1c group (group C, HbA1c ≥6.8% or 51 mmol/mol; n=765). The impact of HbA1c on the Gensini score and the long-term prognosis of CHD with stent implantation were observed. After an average of 1 year of follow-up of the 2,825 patients in a hospital cohort, participants with low or high HbA1c had a higher risk of major adverse cardiac events (MACE) and target lesion revascularization (TLR) compared with participants with moderate HbA1c after adjusting for multiple potential confounders (hazard ratios for low HbAlc, 1.505 and 1.478, respectively, and for high HbAlc, 1.626 and 1.522, respectively). Analysis of HbA1c as a continuous variable showed that each 1% increase of HbA1c was significantly associated with decreased risks of MACE and TLR of 53.5 and 54.2%, respectively, in those with a low HbA1c level and with increased risks of MACE and TLR of 9.5 and 9.2%, respectively, in those with a moderate or high HbA1c level, suggesting a U-shaped association between HbA1c and the risk of MACE and TLR. In conclusion, HbA1c levels, either as a continuous variable or a categorical variable, have a U-shaped correlation with MACE and TLR in CHD patients with stent implantation, even after adjustment for multiple confounders.

## Introduction

At present, coronary heart disease (CHD) is a serious concern in China. Diabetes is considered as harmful to health as CHD, and glycosylated hemoglobin A1c (HbA1c) is an important indicator for monitoring blood glucose levels (1–3). HbA1c can indicate the average blood glucose concentration and is highly reproducible and responsive to minor degrees of abnomality in glucose tolerance. Studies have shown that high HbA1c levels can increase the incidence of CHD and adversely affected its prognosis (4,5). Ueda *et al* found that the levels of HbA1c were significantly higher in patients with major adverse cardiac events compared to those without (4). Fatima *et al* revealed that HbA1c is a reliable predictor of coronary artery disease and that the magnitude of perfusion defects, left ventricular dysfunction and the incidence rate of nonfatal myocardial infarction were higher at an HbA1c level >7.3% (6). A study by Rocco *et al* demonstrated that HbA1c was an important predictor of symptomatic hemorrhage following thrombolysis for acute stroke. The results also suggested that hemorrhage following thrombolysis was a consequence of long-term vascular injury rather than of acute hyperglycemia, and that HbA1c may be a more effective predictor than acute blood glucose or a history of diabetes (7). However, the correlation of low HbA1c levels with coronary artery stenosis and prognosis is not yet clear. The present study investigated the correlation between different levels of HbA1c and coronary artery stenosis and evaluated whether HbA1c is associated with the 1-year risk of major adverse cardiac events (MACE) following successful drug-eluting stent (DES) implantation.

## Materials and methods

### Study subjects

A total of 2,825 consecutive patients with CHD who received the first call for drug-eluting coronary stent implantation at the Department of Cardiology, Zhongda Hospital of Southeast University (Nanjing, China) from June 2008 to June 2012 were included in the study. According to HbA1c levels the patients were divided into three groups. These were the low HbA1c (group A, HbA1c ≤5.9% or 41 mmol/mol; n=1,035), medium HbA1c (group B, 5.9%< HbA1c <6.8% or 41< HbAlc <51 mmol/mol; n=1,025) and high HbA1c groups (group C, HbA1c ≥6.8% or 51 mmol/mol; n=765). Exclusion criteria: i) Had received prior percutaneous coronary intervention or coronary artery bypass surgery; ii) simultaneously implanted bare-metal stents and drug-eluting stents; iii) acute coronary syndrome (ACS); iv) severe anemia and other blood system disease, severe infection, trauma, malignancy, severe liver and kidney dysfunction. Ethical approval was obtained from the Ethics Committee of the Faculty of Medicine, Southeast University (Nanjing, China); all patients provided written informed consent prior to participation in the current study. The study complied with the Declaration of Helsinki.

### Procedure

Criteria for coronary stenting and coronary artery stenosis were assessed. All patients underwent coronary angiography, and the angiography results were judged by one or two experienced specialist physicians. Any main branch with coronary artery stenosis ≥70% underwent coronary stent implantation. The Gensini scoring system was used to quantify the degree of coronary artery stenosis (8): 1 point, ≤25%; 2 points, 26–50%; 4 points, 51–75%; 8 points, 76–90%; 16 points; 91–99%; 32 points, 100%. This stenosis score multiplied by the appropriate factor for the coronary segment provided the branch score. Each patient’s Gensini score was the sum of the scores for every branch.

### Clinical follow up

Follow-up via phone, outpatient and/or readmission, and the 1-year MACE rate were recorded during hospitalization and discharge. MACE includes all-cause mortality, non-fatal myocardial infarction (MI) and target lesion revascularization (TLR, involving thrombolysis, stents or bypass).

### Statistical analysis

Values are expressed as means ± standard deviation. Categorical variables were compared using the Chi square test. Differences in the mean values between two groups were compared using an unpaired t-test or a Wilcoxon rank-sum test. The Kaplan-Meier technique was used to plot cumulative event-free estimates. The Cox proportional hazards model was used to analyze the cumulative survival rate among the groups; differences between groups were assessed with the log-rank test. The Backwald method was used to determine the independent risk factors that will influence clinical outcomes. P<0.05 was considered to indicate a statistically significant result. Statistical analyses were performed using the SPSS software package, version 19.0 (IBM, Armonk, NY, USA).

## Results

### Baseline characteristics

The baseline characteristics are shown in Table I for all 2,825 patients with stent implantation according to the baseline HbA1c level (group A: ≤5.9%, group B: 5.9%< HbA1c <6.8%; group C: ≥6.8%) and by MACE follow up (MACE or no-MACE). The mean duration of follow-up was 327±86.2 days. Family history of CHD, arrhythmia history, chronic kidney disease, cerebrovascular disease, obesity and dual anti-platelet time were similar for the three HbA1c groups. Age, male gender, smoking, hypertension, pulse rate, hemoglobin level, HbA1c level, hyperlipidemia, diabetes and history of heart failure differed among the three HbA1c groups. Hyperlipidemia and diabetes were different between the two MACE groups, whereas the other baseline characteristics were similar between the two MACE groups.

The baseline angiographic characteristics of the study group are summarized in Table II. Gensini score, number of lesions and incidence of multi-vessel disease differed among the three HbA1c groups. The other baseline angiographic characteristics were similar in the three HbA1c groups. Gensini score, number of lesions, number of stents, number of target vessels, incidence of multi-vessel disease, multi-stent use, overlapping stents and ostial lesions were different between the two MACE groups, whereas the other baseline angiographic characteristics were similar in the two MACE groups.

### Survival analysis

**Figure 1:**
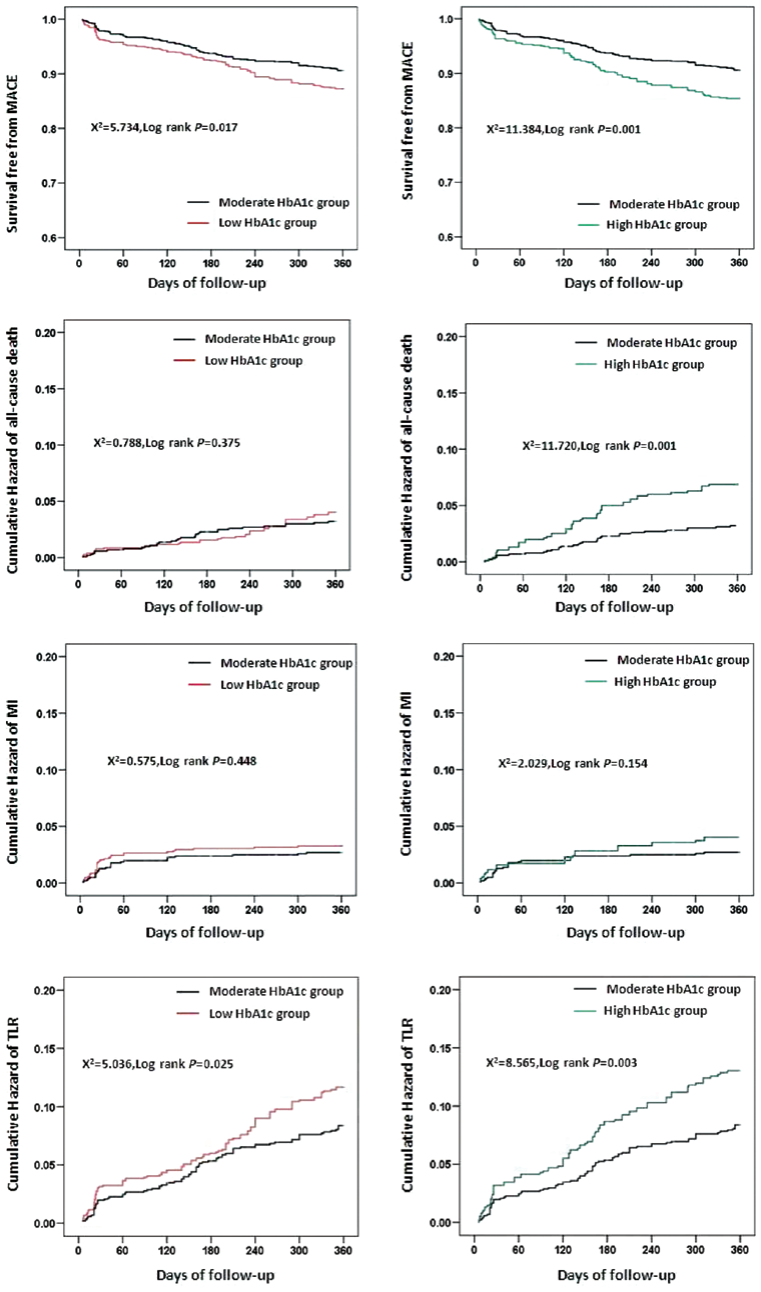
Kaplan-Meier curves representing survival rate free from MACE, and cumulative hazard of all cause mortality, MI and TLR in the three groups. MACE, major adverse cardiac events; Hb1Ac, glycosylated hemoglobin; MI, myocardial infarction; TLR, target lesion revascularization.

As shown in Fig. 1, Kaplan-Meier analysis demonstrated a significantly higher survival rate free from MACE in patients with moderate HbA1c levels (P<0.01, log-rank test). The risk of all-cause mortality of group C was significantly higher than that of group B. The TLR risks of group A and group C were significantly higher than that of group B, while group A and group B, and group B and group C exhibited no significant differences in the risk of MI.

### Correlation analysis

Table III shows that a high or low HbA1c level significantly predicted MACE and TLR after adjusting for age, gender, smoking, hypertension, heart rate, hemoglobin, hyperlipidemia, diabetes, heart failure, Gensini score, number of lesions, number of stents, number of target vessels, multi-vessel disease, multi-stent, overlapping stents and ostial lesions (high HbA1c level, hazard ratio 1.626 and 1.522, respectively; low HbA1c level, hazard ratio 1.505 and 1.478, respectively). A high HbA1c level significantly predicted all cause mortality after adjusting for age, gender, smoking, hypertension, heart rate, hemoglobin, hyperlipidemia, diabetes, heart failure, Gensini score, number of lesions, number of stents, number of target vessels, multi-vessel disease, multi-stent, overlapping stents and ostial lesions (hazard ratio 2.008). Both low and high HbA1c levels are indicated to be risk factors of all-cause mortality, MI and TLR; however, the results are not statistically significant. Analysis of HbA1c as a continuous variable showed that each 1% increase of HbA1c was significantly associated with a decreased risk of MACE and TLR by 53.5 and 54.2%, respectively, in those with a low HbA1c level, and with an increased risk of MACE and TLR by 10 and 9.2%, respectively, in those with a moderate or high HbAlc level, suggesting a U-shaped association between HbA1c and the risk of MACE and TLR, even after adjusting for baseline potential confounders.

## Discussion

In this study of 2,825 older patients with CHD in a hospital cohort followed up for an average of 1 years, an approximately U-shaped association between HbA1c and MACE and TLR was found. Patients with low (≤41 mmol/mol or 5.9%) or high (≥51 mmol/mol or 6.8%) HbA1c levels had a risk of MACE or TLR that was higher than that of participants with moderate (41–51 mmol/mol or 5.9–6.8%) HbA1c levels after adjusting for multiple potential confounders (hazard ratios for low HbAlc, 1.505 and 1.478, respectively, and for high HbAlc, 1.626, 1.522, respectively). Analysis of HbA1c as a continuous variable showed that each 1% increase of HbA1c was significantly associated with decreased risks of MACE and TLR of 53.5 and 54.2%, respectively, in patients with a low level of HbAlc and with increased risks of MACE and TLR of 9.5 and 9.2%, respectively, in those with a moderate or high HbA1c level, suggesting a U-shaped association between HbA1c and the risk of MACE and TLR.

A number of studies have focused on the use of HbA1c levels for the prognosis of patients with diabetes (6,7,9). A meta-analysis of four randomized clinical trials (10), Action to Control Cardiovascular Risk in Diabetes (ACCORD) (11), Action in Diabetes and Vascular Disease: Preterax and Diamicron Modified Release Controlled Evaluation (ADVANCE) (12), the UK Prospective Diabetes Study (13) and Veterans Affairs Diabetes Trial (14), found that all-cause and cardiovascular mortality did not increase in the intensive glycemic control group (target glycated hemoglobin ≤6.5%, fasting blood glucose <6.0 mmol/l or absolute HbA1c reduction 1.5%) compared with a non-intensive control group, with a median follow-up period from 3.4–5.6 years. The External Peer Review Program (EPRP) study of a cohort of 5,815 heart failure patients without diabetes found a U-shaped association between HbA1c and all-cause mortality (15). Xu *et al* found that higher and lower HbA1c levels increased the risk of mortality compared with that for moderate glycemic control (7.1–7.8%), even after adjustment for potential baseline confounders. In comparison with patients with HbA1c levels of 7.5–8.4%, those with lower (<48 mmol/mol, 6.5%) or higher (>69 mmol/mol, 8.5%) HbA1c levels had increased stroke mortality risks of 112% and 143%, respectively (16).

However, the relationship between HbA1c and CHD prognosis remains controversial. Ciciek *et al* (17) found that HbA1c is an independent predictor of the in-hospital mortality of ST elevation MI (STEMI) patients treated with PCI. Corpus *et al* (18) demonstrated that HbA1c is a significant predictor of MACE, target vessel revascularization (TVR) and cardiac death 1 year following PCI in non-diabetic patients. Hadjadj *et al* (19) found that HbA1c was not associated with STEMI prognosis in a small population. Lemesle *et al* (20). demonstrated that HbA1c is not a predictor for cardiac events in diabetic patients with coronary artery disease. The data in the present study show that, compared with patients with moderate HbAlc levels (range, 5.9–6.8%), those with higher HbA1c levels had higher risks of MACE and TLR of 62.6 and 52.2%, respectively. The conflicting relationship between HbA1c levels and prognosis may have several interpretations. One reason may be due to different definitions and follow-up times of the end point. Another reason may be differences in the study. Corpus, *et al* (18) defined the primary endpoint as TVR, secondary endpoint as cardiac death, MI, recurrent angina, stroke, congestive heart failure, renal failure and cardiac rehospitalization, and the study was of non-diabetic patients with CHD. Lemesle *et al* (20) defined the primary endpoint as mortality, MI and TVR, used a 1-year follow-up, ~70% of patients received stents and studied CHD patients with DM. In the present study, the endpoint is defined as mortality, non-fatal MI and TLR through PCI, and the subjects were CHD patients with or without DM; all patients received stent implantation. HbA1c and a composite endpoint (cardiovascular death, nonfatal MI or TLR) to assess whether the definition of the end result affected the correlation between HbA1c and the endpoint. Cardiovascular death and TLR were found to be significantly correlated with HbA1c.

There have been relatively few studies concerning the correlation between low HbA1c level and the prognosis of CHD patients. The present study suggests that compared with patients with moderate HbAlc levels (range, 5.9–6.8%), those with lower HbA1c levels had increased risks of MACE and TLR of 50.5 and 47.8%, respectively. The results are consistent with a U-shaped correlation between HbA1c and the prognosis of diabetic patients. Intensive glucose control, resulting in low levels of HbA1c and severe hypoglycemia, may lead to subsequent higher mortality, which may explain the results of this study.

The present study has several limitations. First, antidiabetic drugs may have affected the results. This study was not designed to test the results of the treatment of diabetes; due to lack of data, it was not possible to study the therapeutic effect of such treatment. Secondly, the HbA1c level measurements were not repeated, and in subsequent years, HbA1c levels may have changed. Thirdly, the observed increases in MACE and TLR were independent of sex, gender, hypertension, hyperlipidemia and history of heart failure. Nonetheless, it is not possible to exclude residual confounding by other known and unknown risk factors, such as fasting blood glucose or inflammatory markers, which were not covered in the analysis. Fourthly, due to telephone interviews and the cause of mortality being reported by a family member or loved one, misjudgment of the cause of mortality cannot be completely ruled out. Finally, the severity of cardiovascular disease at baseline cannot be assessed based on the self-reported history. Therefore, misjudgment of cardiovascular disease cannot be eliminated.

In summary, this study found that HbA1c levels, either as a continuous variable or a categorical variable, have a U-shaped correlation with MACE and TLR in CHD patients with stent implantation, even following adjustment for multiple confounders. Based on the aforementioned limitations of this study, more rigorous and comprehensive studies are required to confirm these findings.

## Figures and Tables

**Table I:** Baseline clinical characteristics in all 2,825 patients, grouped by baseline HbA1c and by MACE.

	Groups by baseline HbA1c, %				MACE		
				
Variable	≤5.9	5.9–6.8	≥6.8	P-value	MACE	No-MACE	P-value
Age (years)	59.21±11.17	59.07±11.12	61.02±10.43	<0.001	60.56±10.59	59.53±11.04	0.108
Male (%)	731	456	317	0.003	1689	221	0.266
Smoking (%)	493	456	317	0.038	1106	160	0.138
Hypertension (%)	596	630	526	<0.001	1535	217	0.129
Pulse (bpm)	70.3	70.9	73.19	<0.001	71.52	71.30	0.752
Hemoglobin (g/l)	137.45	137.27	135.56	0.029	136.50	136.93	0.652
Hemoglobin A1c, %	4.49	5.39	7.69	<0.001	5.79	5.78	0.966
Family history of CHD (%)	11	20	16	0.174	44	3	0.156
Arrhythmia (%)	178	156	114	0.335	401	47	0.176
Hyperlipidemia (%)	380	427	342	0.001	995	154	0.025
Diabetes (%)	77	120	384	<0.001	496	85	0.013
Chronic kidney disease (%)	617	595	453	0.809	1472	193	0.241
Cerebrovascular disease (%)	93	72	64	0.234	201	28	0.453
Body mass index (kg/m^2^)	20.0	23.4	24.5	0.376	24.3	23.2	0.144
History of heart failure (%)	8	24	41	<0.001	65	8	0.487
Dual antiplatelet time (days)	197.1	196.8	197.8	0.920	199.01	196.94	0.488

CHD, coronary heart disease; HbA1c, glycosylated hemoglobin; MACE, major adverse cardiac events.

**Table II:** Baseline angiographic characteristics according to baseline HbA1c group and MACE after follow-up.

	Groups by baseline HbA1c, %				MACE		
				
Variable	≤5.9	5.9–6.8	≥6.8	P-value	MACE	No-MACE	P-value
Gensini score	29.09±34.48	28.70±33.69	37.77±38.95	<0.001	40.87±41.49	30.01±34.66	<0.001
Number of lesions	1.40	1.45	1.70	<0.001	1.77	1.46	<0.001
Number of stents	1.94	1.25	1.87	0.394	1.73	1.94	0.020
Number of target vessels	1.56	1.54	1.53	0.776	1.44	1.56	0.005
Multi-vessel disease	240	228	238	<0.001	583	123	<0.001
Multi-stent	581	573	414	0.661	1401	167	0.016
Overlapping stents	298	312	226	0.715	752	84	0.030
Small vessels	67	79	53	0.544	178	21	0.324
Long lesions	511	485	365	0.618	1213	148	0.066
Calcification	93	113	68	0.199	248	26	0.118
CTO	41	47	21	0.132	96	13	0.537
Bifurcation	348	342	251	0.936	834	107	0.308
Ostial lesions	189	188	160	0.290	459	78	0.022
Type C lesions	401	374	277	0.450	2032	270	0.351

CTO, chronic total occlusion; HbA1c, glycosylated hemoglobin; MACE, major adverse cardiac events.

**Table III:** Adjusted hazard ratios of MACE, all-cause mortality, MI and TLR in 2,825 patients with CHD following PCI by continuous and grouped HbA1c levels (mean follow-up, 1 year).

	HbA1c continuous^a^,%		HbA1c groups,%		
		
Variable	≤5.9^b^	>5.9^c^	≤5.9	5.9–6.8	≥6.8
MACE					
Crude hazard ratio	0.435 (<0.001)^d^	1.100 (0.004)^e^	1.379 (0.017)^f^	Ref	1.101 (0.084)
Model 1	0.474 (0.098)	1.004 (0.956)	1.483 (0.005)^f^	Ref	1.712 (<0.001)^c^
Model 2	0.465 (0.002)^e^	1.095 (0.01)^e^	1.505 (0.004)^f^	Ref	1.626 (0.001)^b^
Mortality					
Crude hazard ratio	0.324 (0.115)	1.172 (0.034)^f^	1.234 (0.376)	Ref	1.172 (0.034)^a^
Model 1	0.780 (0.104)	0.985 (0.892)	1.246 (0.380)	Ref	2.092 (0.002)^b^
Model 2	0.535 (0.158)	1.049 (0.466)	1.264 (0.354)	Ref	2.008 (0.004)^b^
MI					
Crude hazard ratio	0.128 (0.005)^e^	1.002 (0.989)	1.217 (0.449)	Ref	1.002 (0.989)
Model 1	0.663 (0.033)^f^	1.003 (0.985)	1.359 (0.262)	Ref	1.712 (0.056)
Model 2	0.6 (0.295)	1.116 (0.068)	1.418 (0.202)	Ref	1.582 (0.106)
TLR					
Crude hazard ratio	0.228 (<0.001)^d^	1.127 (0.042)^f^	1.385 (0.026)^f^	Ref	1.127 (0.042)^a^
Model 1	0.577 (0.110)	1.194 (0.864)	1.460 (0.013)^f^	Ref	1.595 (0.004)^b^
Model 2	0.458 (0.003)^e^	1.092 (0.021)^f^	1.478 (0.01)^e^	Ref	1.522 (0.009)^b^

Ref, reference, with the lowest rate. Model 1 adjusted for age, sex, smoking, hypertension, heart rate, hemoglobin, hyperlipidemia, diabetes, heart failure. Model 2 additionally adjusted for Gensini score, number of lesions, number of stents, number of target vessel, multi-vessel disease, multi-stent, overlapping stents and ostial lesions.

a Hazard ratios for HbA1c continuous were per 1% increase;

b HbA1c level ranged from 4.2 to 5.9 in the analysis of cardiovascular disease and stroke mortality;

c HbA1c level ranged from 5.9 to 15.5 in the analysis of cardiovascular disease and stroke mortality;

d P<0.001,

e P<0.01 and

a P<0.05.
